# Genomic characterisation of extensively drug-resistant *Acinetobacter baumannii* isolates from a tertiary hospital in Ghana

**DOI:** 10.1371/journal.pone.0336931

**Published:** 2026-03-17

**Authors:** Isabella E. Ashley-Agbedor, Samiratu Mahazu, Fleischer C. N. Kotey, Jennifer Amedior, Bright Adu, Emmanuel Darko, Paul Kwao, Nicholas T. K. D. Dayie, Anthony S. Ablordey, Japheth A. Opintan

**Affiliations:** 1 Department of Medical Microbiology, University of Ghana Medical School, College of Health Sciences, University of Ghana, Accra, Ghana; 2 Bacteriology Department, Noguchi Memorial Institute for Medical Research, College of Health Sciences, University of Ghana, Accra, Ghana; 3 Immunology Department, Noguchi Memorial Institute for Medical Research, College of Health Sciences, University of Ghana, Accra, Ghana; Universidade Católica Portuguesa Escola Superior de Biotecnologia: Universidade Catolica Portuguesa Escola Superior de Biotecnologia, PORTUGAL

## Abstract

*Acinetobacter baumannii* (*A. baumannii*) is an emerging “superbug” whose infections have become extremely difficult to treat due to its diverse antimicrobial resistance mechanisms and resistance to last-resort antibiotics including carbapenems. Despite this, data on genetic determinants and genomic context of carbapenemase genes in *A. baumannii* are scarce in Ghana. This study investigated the genetic determinants of carbapenem resistance in clinical isolates of *A. baumannii* (*Ab*) and explored the genetic contexts of carbapenemase-encoding genes in extensively drug-resistant *A. baumannii* (XDR-*Ab*). We analysed 65 archived clinical *A. baumannii* isolates. Identification and antimicrobial susceptibility profiles of the isolates were determined using a MALDI-TOF-MS and the Microscan device, respectively. Carbapenem resistant *A. baumannii* (CR-*Ab*) isolates were screened for carbapenemase-encoding genes (*bla*_*NDM-1*_*, bla*_*KPC*_*, bla*_*VIM*_*, bla*_*OXA-48*_*, bla*_*OXA-23*_*, bla*_*OXA-58*_*,* and *bla*_*IMP*_*)* using loop-mediated isothermal amplification (LAMP). Six XDR-*Ab* isolates were whole-genome sequenced (WGS) using Nanopore MinION. Carbapenem resistance was observed in 18/65 (27.7%) isolates. All CR isolates were resistant to penicillins, cephalosporins, fluroquinolones, aminoglycosides, sulfonamides and carbapenems (minimum inhibitory concentrations, MICs, > 2µg/ml - > 8µg/ml). The most predominant resistance gene, *bla*_*NDM-1*_ (33.3%**)**, was found to co-exist with *bla*_*OXA-23*_ (27.8%) or *bla*_*OXA-58*_ (16.7%), while *bla*_*OXA-420*_ (12.5%) was the least prevalent gene detected. The sequence types identified among the isolates were ST2 (33.3%), ST164 (16.7%), ST214 (16.7%), ST52 (16.7%), and ST16 (16.7%) according to MLST-Pasteur scheme. The study highlights the carriage of multiple carbapenemase genes, other AMR-encoding genes and efflux pumps in XDR clinical *A. baumannii* isolates. To the best of our knowledge, the study reports for the first time, the detection of ST2 OXA-23, NDM-1 and ST164 OXA-58, NDM-1-producing *A. baumannii* strains at the Korle Bu Teaching Hospital. These strains belong to high-risk clones and continuous surveillance through molecular epidemiological studies and public health interventions are urgently needed to control their spread in Ghana.

## Introduction

*Acinetobacter baumannii* is an emerging notorious nosocomial pathogen that has frequently been associated with hospital-acquired infections such as septicemia, urinary tract infections, wound infections, ventilator-associated pneumonia, and surgical site infections, with 20–72.5% of these infections occurring in intensive care units (ICUs) [1–6]. However, the management of these infections continues to be exceedingly difficult because of the wide spectrum of antimicrobial resistance of the organism [7]. Recently, multidrug-resistant *A. baumannii* (resistance to at least one antimicrobial agent in three or antimicrobial categories) (MDR-*Ab*), and/or extensively drug-resistant *A. baumannii* (XDR-*Ab*) strains (resistance to one agent in all but two or less antimicrobial class) have been implicated in several infections in healthcare facilities worldwide [1,8–17]. Whereas in 2017, CR-*Ab* was the Number one critical pathogen, in 2024, CR-*Ab* remains one of the critical pathogens on World Health Organization (WHO) priority lists for which novel therapeutic antibiotics are urgently needed [18–20]. CR in *A. baumanni* is chiefly mediated by the acquisition of carbapenemases through horizontal gene transfer, mostly facilitated by mobile genetic elements (MGEs) [21–23]. Several studies have reported the production of Class D beta-lactamases (CHDLs) *OXA*-23-like, *OXA24*/40-like, *OXA*-58-like, *OXA*-143-like, and *OXA*-235-like enzymes as common genetic determinants of CR in *A. baumannii*, although the carriage of *bla*_*NDM-1*_, *bla*_*VIM*_*,* and *bla*_*IMP*_ genes has also been extensively described in CR-*Ab* strains worldwide [24–28]. Across Africa, *NDM*-1, *OXA*-23*,* and *OXA*-58 are the most prevalent carbapenemases reported [11,29–34]. In Ghana, since 2019, when the first report of *NDM-1-*producing *A. baumannii* was released [19], limited studies have also described clinical *A. baumannii* strains harboring *bla*_*OXA-23*_, *bla*_*NDM-1*_ and *bla*_*OXA-58*_ genes from Ghanaian hospitals [12,35,36]. Molecular characterisation of CR-*Ab* isolates has demonstrated the significant role of MGEs (conjugative plasmids, insertion sequences, transposons, and integrons) in the global spread of carbapenemases [22,37–42]. Nevertheless, in Ghana, the paucity of molecular data highlighting the resistance and dissemination mechanisms of CR*/*XDR*-Ab* undermines efforts to develop effective and efficient strategies for combating infections caused by these strains. In this study, we investigated the genetic determinants of CR as well as the genetic context of carbapenemase-encoding genes and their association with MGEs in whole genome sequenced XDR-*Ab* isolates from KBTH.

## Methods

### Study design and setting

The study analyzed a total of 65 archived non-duplicate *A. baumannii* isolates recovered from in-patients’ specimens processed for culture and susceptibility testing at. the KBTH Central Laboratory between June and December 2022. The KBTH is the largest referral centre in Ghana and has 21 specialised clinical and diagnostic departments. The isolates were stored in nutrient broth with 10% glycerol and kept in a −80 ^0^C freezer. Anonymized data and bacterial isolates were accessed on the July 20, 2023, to obtain information on sample types from which the isolates were recovered and the wards of origin of isolates solely for analysis purpose. Authors had no access to information that could identify individual patient.

### Identification of isolates

The stored cultures were brought to room temperature to thaw and subsequently sub-cultured on nutrient agar (Beckton, Dickinson and Company, USA) and incubated aerobically for 24 hours at 37 ^0^C. Discrete colonies were selected and identified using the MALDI-TOF Biotyper (Bruker Daltonics, Karlsruhe, Germany).

### Antibiotic Susceptibility Testing

The susceptibility profiles of isolates to meropenem (10 µg), imipenem (10 µg), and doripenem (10 µg) (BD BBL, USA) were determined by the Kirby-Bauer disc diffusion method as described in the CLSI [43]. For quality control, a known carbapenem susceptible strain *E. coli* ATCC 25922 and carbapenem producing *A. baumannii* NCTC 13304 were used. Isolates that were found to be resistant to imipenem, doripenem and meropenem (≤15 mm zone of inhibition) were further tested to determine their MICs for 14 antibiotics including meropenem, doripenem and imipenem using the Microscan (Beckman Coulter, USA). Interpretation of MIC results was done following the CLSI guidelines (2023) [43]. *A. baumannii* isolates that were resistant to all the carbapenems tested (meropenem, doripenem and imipenem) were selected for genotypic characterization.

### Genotypic detection of carbapenemase genes

Genomic DNA extraction of all CR isolates was performed using the MagAttract High Molecular Weight (HMW) DNA Kit (Qiagen, Hildon, Germany), according to the manufacturer’s instructions [44]. LAMP reaction was used to screen for carbapenemase-encoding genes *bla*_*NDM-1*_, *bla*_*KPC*_, *bla*_*VIM,*_ *bla*_*OXA-48*_, *bla*_*OXA-23*_, *bla*_*OXA-58*_ and *bla*_*IMP*_ using primers described by Lahiri and colleagues [45].

### Whole-genome sequencing and multi-locus sequence typing

Whole-genome sequencing was performed on six XDR-*A. baumannii* isolates. Genomic DNA was extracted from the isolates using a NucleoSpin tissue kit according to the manufacturer’s instruction (Macherey-Nagel, Düren, Germany) [46]. The Native Barcoding Kit EXP-NBD104 and the ligation sequencing kit SQK-LSK109 enabled the preparation of gDNA libraries for long-read sequencing [47]. Sequencing was performed using the Nanopore MinION (Oxford Nanopore Technologies, United Kingdom) [48] and sequences were assembled with Flye v2.6 [49]. MLST of the isolates was performed using the online tool PubMLST Pasteur scheme [50].

### Bioinformatics, phylogenetic, and statistical analyses

The online system RAST and BLAST tools were used to annotate and compare the assembled sequences [35]. Resfinder v4.3.3 (Center for Genomic Epidemiology) [51] was used to detect AMR-encoding genes. Mobile genetic elements (MGEs) were identified using mge_finder 1.1.2, Uniprot BLAST and ISfinder (www-is.biotoul.fr) [52]. The SnapGene viewer v4.1.9 and Easyfig v2.2.5 were used to visualise the genetic contexts of carbapenemase-encoding genes and compare sequences [35,53]. To determine the genetic relatedness of isolates, a single nucleotide polymorphism (SNP) based phylogenetic tree was constructed using the CSIPhylogeny tool (Center for Genomic Epidemiology). *A. baumannii* reference strain GCF_0086326351.1 was used as rooting genome [54]. Figtree V1.4.4 enabled the visualisation and annotation of phylogeny trees [54,55]. The isolates chromosomes and plasmid sequences have been deposited with GenBank (SAMN43561363, SAMN43561408, SAMN43561092, SAMN43800676 and SAMN43303015).

Statistical Products and Services Solutions (SPSS), version 22.0 for Microsoft Windows [56], was used to analyze data, which involved descriptive statistics (such as means, tables, and graphs) and Chi-square tests at 95% confidence interval (CI). A p-value of ≤ 0.05 was considered statistically significant.

### Ethical approval

The study protocol was reviewed and approved by the Korle Bu Teaching Hospital Scientific and Technical Committee/Institutional Review Board (KBTH-STC/1RB/00071/2023). Data and bacterial isolates used for the study were anonymized, hence informed consent was not required.

## Results

### Antimicrobial resistance profile of CR isolates

The 65 *A. baumannii* isolates, were recovered from wound swabs (*n* = 26), urine (*n* = 23), blood (*n* = 7), aspirates (*n* = 4), catheter tip (*n* = 2), HVS (*n* = 2) and eye swab (*n* = 1). Overall, CR was observed in 18/65 (27.7%) isolates. Table 1 shows the MICs of CR-isolates. CR isolates were mostly isolated from surgical wounds (n = 7, 44.4%), urine (n = 7, 38.9%), blood (n = 2, 11,1%), ulcer wounds (n = 1, 5.5%) and aspirate (n = 1, 5.5%).

**Table 1:** Antimicrobial resistance profile of CR-*A. baumannii* isolates.

Antibiotics		MIC (ug/mL)	
	MIC_50_	MIC_90_	% Resistance
Ampicillin	>16	>16	100
Ampicillin/sulbactam	>16/8	>16/8	100
Cefepime	>16	>16	100
Cefotaxime	>32	>32	100
Ceftazidime	>16	>16	100
Cefuroxime	>16	>16	100
Ciprofloxacin	>2	>2	100
Levofloxacin	>4	>4	72
Tobramycin	>8	>8	83
Amikacin	>32	>32	67
Gentamycin	>8	>8	83
Meropenem	>8	>8	100
Imipenem	>8	>8	100
*Trime/sulfame	>2/38	>2/38	89

*Trime/sulfame: Trimethoprim/sulfamethoxazole.

### Genotypic detection of carbapenemase genes in CR-*A-baumannii* isolates

All 18 CR-*Ab* isolates screened for carbapenemase-encoding genes harbored a corresponding *bla*_*OXA-51*_ *carbapenemase* gene. Additionally, the Metallo beta-lactamase (MBL) *bla*_*NDM-1*_ (85.7%) and CHDL *bla*_*OXA-23*_ (71.4%) were the most prevalent genes, followed by *bla*_*OXA-58*_ (42.9%) and *bla*_*OXA-420*_ (14.3%) was the least prevalent gene detected. Four (22.2%) isolates lacked any acquired carbapenemases.

### Whole genome sequencing of XDR-*A. baumannii* isolates

Table 2 shows the genomic characteristics of XDR-*A. baumannii* isolates. The most predominant gene was *bla*_*NDM-1*_ (66.7%) detected in isolates Aba05, Aba08, Aba20 and Aba22 and coexisted with either *bla*_*OXA-23*_ (Aba05 and Aba20) or *bla*_*OXA-58*_ (Aba08 and Aba22). Besides, the isolates harbored other AMR encoding genes that conferred resistance against aminoglycosides (aac (6’) aadA2, aph (3’)-19, 16SrRNAmethythransrase), fluoroquinolones (*A. baumannii* ParC/gyrA), sulfonamides (Sul1/Sul2), disinfectants/antiseptics (qacE delta1/qacG) and macrolides (macrolide phosphor-transferase, mphE). Multidrug efflux pumps, including members of the major facilitator superfamily (MFS) and resistance–nodulation division family (RND) were also identified in the isolates’ genomes.

**Table 2:** Molecular characteristics of XDR- *A. baumannii* isolates.

Isolate ID	Sample type	*bla*_*OXA-51-*_ *like*	Carbapenem gene	Location	Other AMR-genes	Sequence types
Aba05	wound	*bla*_*OXA-66*_	*bla*_*OXA-23*_ *bla*_*NDM-1*_	Chromosome Chromosome	*armA, ParC, gyrA* *Sul2, tet (b), msrE/mphE*	ST2
Aba08	wound	*bla*_*OXA-66*_	*bla*_*OXA-58*_ *bla*_*NDM-1*_	Plasmid Plasmid	*ANT (3”)-ll, APH (3’), APH (3’), aac (3) -llc, ParC, gyrA, Sul2, msrE*	ST16
Aba11	blood	*bla*_*OXA-98*_	*bla*_*OXA-58*_ *bla*_*NDM-1*_	Plasmid Chromosome	*ANT (3”)-ll, APH (3’), APH (3”), aac (3)-llc, ParC, gyrA Sul2, Tet (39)*	ST52
Aba20	shunt	*bla*_*OXA-66*_	*bla*_*OXA-23*_ *bla*_*NDM-1*_	Chromosome	*armA, APH (3’), ParC, gyrA, Tet (R), Sul1/2, msrE/mphE*	ST2
Aba22	urine	*bla*_*OXA-91*_	*bla*_*OXA-58*_ *bla*_*NDM-1*_	Plasmid	*armA, ANT (3”)-19, aadA2, aac (6’)-ld4, aar-3, ParC, gyrA Sul1, msrE/ mphE*	ST164
Aba23	urine	*bla*_*OXA-378*_	*bla*_*OXA-420*_	Plasmid	*APH (3)-lb, APH (6)-ld, APH (3’) la, ANT (2’)-la, ParC, gyrA msrE/mphE* *Tet (39)*	ST2146

### Genetic contexts of *bla*_*OXA-23*_*, bla*_*NDM-1*_*, bla*_*OXA-58*_ and *bla*_*OXA-420*_

**Fig 1:**
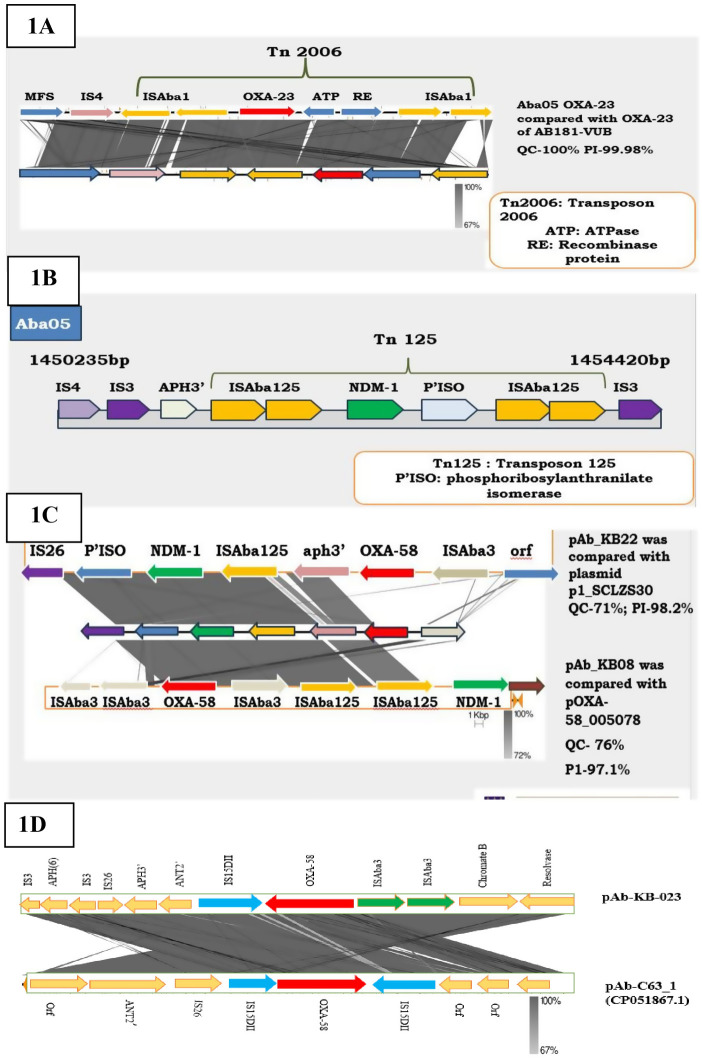
(A–D). The genetic contexts of *bla*_*OXA-23*_*, bla*_*NDM-1,*_ *bla*_*OXA-*58_ and *bla*_*OXA-420*_ in CR-*A. baumannii.* (A) Shows *bla*_*OXA-23*_ genetic context in Aba05 and Aba20 compared with *A. baumannii* strain AB181-VUB, *bla*_*OXA-23*_ gene was embedded in transposon Tn2006. (B) In isolates Aba05 and Aba20, the *bla*_*NDM-1*_ genes were identified between chromosomes sequences (1450235 bp - 145440 bp) and (838,300 bp – 843,418 bp) respectively, captured by transposon Tn125. (C). The whole plasmid sequence of Aba08 and Aba22 encoding *bla*_*OXA-58*_ and *bla*_*NDM-1*_ genes compared with plasmids pOXA-58_005078 (D) shows *bla*_*OXA-420*_ surrounding regions identified in Aba23 plasmid compared with plasmid pAb-c631_1.

Fig 1 (A–D) depicts the genetic environments of carbapenemase genes detected in the study isolates. The *bla*_*OXA-23*_ in Aba05 and Aba20 was chromosomally embedded and was flanked upstream by *TetB*-IS4-element-ISAba1-ISAba1 and downstream by ISAba1-ISAba1. Furthermore, composite transposon Tn2006 was detected in both isolates (Fig 1A).

The *bla*_*NDM-1*_ was also detected on the chromosomes of Aba05, Aba11 and Aba20. In Aba20, the *bla*_*NDM-1*_ was flanked by two copies of ISAba125 upstream and, a sequentially arranged ISAba125-ISAba125-IS3-ISAba14 elements downstream, embedding in a composite transposon Tn125 as described by Poirel et al (2012) (fig 1B). A further screening of the upstream region of *bla*_*NDM-1*_ showed an array of *aph3-*ISAba33-IS4-IS3-element. However, in Aba20, only one copy of ISAba125 was detected upstream of *bla*_*NDM-1*_ and ISAba34 downstream. Also, IS4 and *aph3*’ were located upstream of ISAba125. In Aba11, an entirely different genetic environment was observed for *bla*_*NDM-1*_ in which insertion sequence ISAde1 bracketed the gene. Flanking ISAde1 downstream*,* were the sequentially arrayed *aph3’,* ISAba14 and IS3 transposase elements.

Plasmid-borne (pAb_KB08 and pAb_KB22) *bla*_*NDM-1*_ and *bla*_*OXA-58*_ was carried by isolates Aba08 and Aba22. In the genetic environment of *bla*_*NDM-1*_ and *bla*_*OXA-58*_ on plasmid pAb_KB08, the presence of msr, mph2’, resolvase-invertase-type recombinase ISAba3, ISAba3, *bla*_*OXA58*_, ISAba3, ISAba125, ISAba125, *bla*_*NDM-1*,_ *ble*_*MBLs,*_ and ISAba32 were detected. A nucleotide BLAST analysis of the whole plasmid showed it shared 98.17% similarities with plasmid p1_SCLZS30, (query cover 71%; accession number: CP090385.1) and 97.1% identity with plasmid pOXA-58_005078 (query cover 76%; accession number: CP033131.1) (Fig 1C). Although the *bla*_*OXA-66*_ gene was detected in both isolates, no insertion sequences were detected upstream or downstream of the gene. However, the genetic environment of *bla*_*OXA-58*_ in isolate Aba11 varied from that of Aba08 and Aba22. For Aba11, insertion sequence IS15DII and ISAba3-*aph3*’ were found upstream and downstream of *bla*_*OXA-58*_ respectively. A BLAST search of this plasmid showed 63% query coverage and 99.93% percentage identity with plasmid pNDM_SCLZS86 (accession number: CP041590.1).

The *bla*_*OXA-420*_ (an OXA-58-like enzyme) was also plasmid-encoded carried by isolate Aba23 (pAb_KB23). Insertion sequences IS15DII and ISAba3 flanked the *bla*_*OXA-420*_ upstream and downstream respectively. In the immediate upstream region of IS15DII, an array of IS26, *aph(3’)-la, aph(3”)-b, aph(6)-Id and ant(2”)-la* sequentially arranged, whilst in the downstream of *bla*_*OXA-420*_*, mph(E)*, *Sul2* and *tet (39)* were detected. In addition, other genetic elements such as ISAlw27, IS1006 and ISAcsp12 were also identified in the upstream region of IS26. A nucleotide BLAST analysis of plasmid pAb_KB23 showed, its 99.74% identity with plasmid pAb-C63_1 (100% query cover; accession number CP051867.1) (Fig 1D).

### Genetic diversities of XDR-*A. baumannii* isolates

**Fig 2:**
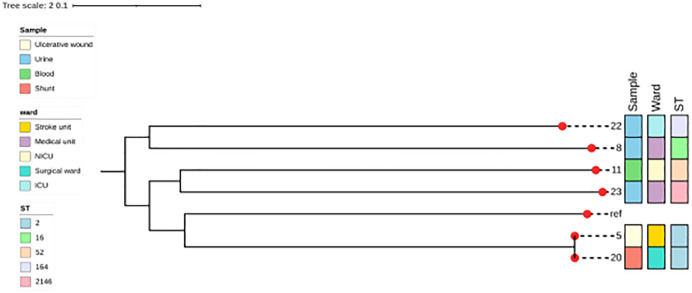
Genetic relation of XDR-*A. baumannii* isolates recovered from different in-patient specimens and their sequence types (STs).

Fig 2 shows the phylogeny of XDR isolates. The isolates were genetically diverse belonging to sequence types, ST 2 (Aba20 and Aba05), ST 164 (Aba22), ST 2146 (Aba23), ST 52 (Aba11), and ST 16 (Aba08). Nevertheless, isolates Aba05 and Aba20 recovered from two patients admitted to the surgical and stroke units respectively, belonged to the same sequence type ST 2,

## Discussion

MDR*-A. baumannii* infections continue to pose a major threat in healthcare settings, yet much remains to be understood and discovered about this nosocomial pathogen. In the current study, we report approximately a 28% prevalence of CR-*Ab* in a major referral hospital in Ghana. Comparatively, this prevalence is higher than the 5.6% reported for the same country by Ayibieke et al [35], indicating a potential increase in the prevalence of CR-*Ab*. Our findings aligns with CR-*Ab* prevalence of 20% and 22.4% reported from some studies conducted in the USA and Canada respectively [57,58]. Elsewhere in Africa, higher prevalence of 61% and 49% have been reported in Ethiopia and Algeria respectively [29,59]. The rising prevalence of CR-*Ab* across the Sub-Saharan African region could further worsen the disease burden and mortality associated or attributed with antimicrobial resistance (AMR) [60].

In *A. baumannii,* the genetic basis of carbapenem-resistance has widely been associated with the production of carbapenem-hydrolyzing class D β-lactamases and Metallo β-lactamases [61]. This observation is consistent with our findings where most of the isolates harbored Class B (NDM-1) and Class D (OXA-23, OXA-58 and OXA-420) carbapenemases.

We observed the co-existence of *bla*_*NDM-1*_ with either *bla*_*OXA-23*_ or *bla*_*OXA-58*_ in XDR-*Ab* isolates. The emergence of *A. baumannii* strains co-harboring *bla*_*OXA-23*_ and *bla*_*NDM-1*_ was first reported in India, a sub-continent considered to be the reservoir for OXA-23 and NDM-1-producing *A. baumannii* strains [62]. Subsequently, Nepal, Egypt, Pakistan and Libya have also reported the co-existence of *bla*_*OXA-23*_ and *bla*_*NDM-1*_ in *A. baumannii* [16,28,61,63]. Our observation of the co-existence of *bla*_*NDM-1*_ and *bla*_*OXA-23*_ is consistent with a previous study in Ghana that highlighted the predominance of CR-*Ab* co-harboring *bla*_*OXA-23*_ and *bla*_*NDM-1*_ [12]. Studies in South Africa, however, show that CR-*Ab* isolates predominantly carry only the *bla*_*OXA-23*_ gene. [33,64,65]

Analysis of the genetic environment of *bla*_*NDM-1*_ and *bla*_*OXA-23*_ revealed the presence of ISAba1 and ISAba125 upstream of both genes respectively. The insertion of ISAba1 and ISAba125 upstream of *bla*_*OXA-23*_ and *bla*_*NDM-1*_ respectively, provided strong sequence promoters and resulted in increased carbapenem resistance among *A. baumannii* isolates [25,66–68]. More importantly, *bla*_*OXA-23*_ and *bla*_*NDM-1*_ resided within composite transposons Tn2006 and Tn125 respectively which are major dissemination vehicles for *bla*_*OXA-23*_ and *bla*_*NDM-1*_ genes worldwide [24,25,67]. The detection of Tn2006 and Tn125 in our isolates raises concern since these MGEs could further facilitate the spread of *bla*_*NDM-1*_ and *bla*_*OXA-23*_ genes among *A. baumannii* strains and other Gram-negative pathogens across the hospital.

The study also identified four distinct plasmids (pAbKB_08, pAbKB_11, pAbKB_22 and pAbKB_23) encoding *bla*_*OXA-58*_*, bla*_*NDM-1*_ and *bla*_*OXA-420*_ genes. The carriage of plasmid-borne carbapenemase-encoding genes by isolates recovered from patients admitted to different wards, buttresses the disperse nature of these transferable MGEs among *A. baumannii* isolates across the hospital. This confirms *A. baumannii* and other Gram-negative organisms’ propensity to easily acquire and accumulate AMR-genes [69,70]. In 2003, infection outbreaks caused by OXA-58-producing *A. baumannii* was first reported from France, and are currently on a global scale [71–75]. However the occurrence of CR-*Ab* harboring plasmid-encoded *bla*_*OXA-58*_ and *bla*_*NDM-1*,_ is scarce [70], limited studies from Thailand, Malaysia and China, have described plasmid-borne *bla*_*OXA-58*_ and *bla*_*NDM-1*_ in *A. baumannii*, *A. pitti* and *A. nosocomialis* [76–78]. In Ghana, whereas a plasmid-borne *bla*_*OXA-58*_, was described in CR-*Ab* [35], to the best of our knowledge this study is the first to report plasmids co-harboring *bla*_*OXA-58*_ and *bla*_*NDM-1*_ in this country. Moreover, plasmid pAbKB_22 99.80% similarities with plasmid GD03255 identified in *A. bereziniae* and its 98.2% identity with plasmid pl_SCLZS30 carried by *A. towneri,* as well as plasmid pAbKB_08 97.61% identity with plasmid pOXA-58_010062 harbored by *A. wuhouensis* strain*,* (all detected in China, unpublished), may seem to point to the fact that the occurrence of plasmid-borne *bla*_*OXA-58*_ and *bla*_*NDM-1*_ in *Acinetobacter species* may be endemic in Asia. Similar to our findings, a recent report from Brazil also identified plasmid-borne *bla*_*OXA-58*_ and *bla*_*NDM-1*_ in *A. baumannii* clinical isolates [70]. This gives indication of the possible steady spread of these MGEs across the nations.

CR-*Ab* carrying *bla*_*OXA-420*_ was first detected in Nepal [78,79] and subsequent studies in Nigeria and Kenya have also reported on OXA-420-producing *A. baumannii* isolates [80,81]. In Ghana, two studies have described OXA-420 *A. baumannii* producers [12,35] and this study is the third to report on these strains. Interestingly, plasmid pAbKB_23, detected in the current study share 100% sequence identity with plasmid pAb-c63, previously described by Ayibieke et al in Ghana [35]. The detection of *bla*_*OXA-420*_ in three separate studies conducted in three different regions (Accra, Takoradi and Eikwe) in Ghana affirms the suspicion of Ayibieke et al of the probable circulation of *bla*_*OXA-420*_ in hospitals in Ghana requiring urgent infection control and prevention measures to stop their spread.

Globally, outbreaks of infections due to diverse populations of *A. baumannii* strains have been reported and the majority of these infections are driven by global clone 1 (GC1) and global clone 2 (GC2), represented as ST1 and ST2 respectively. ST2 is the dominant sequence type of *A. baumannii* in Thailand and most widespread clones, influencing the spread of carbapenem-resistance genes around the world. [25,82,83]. ST2 *A. baumannii* strains are also noted for XDR and treatment difficulties or failures due to their higher virulence [84]. In our study, the two ST2 isolates (Aba05 and Aba20) were genetically related, indicating a possible clonal spread. In Ghana, whereas ST1 *A. baumannii* isolates have been previously described in two separate studies [35,36], to the best of our knowledge, this is the first detection of ST2 *A. baumannii* isolates in Ghana. Studies conducted in Lebanon, Greece, Italy, Turkey and Denmark have associated a series of nosocomial infection outbreaks with ST2 *A. baumannii* isolates carrying *bla*_*OXA-23*_ and *bla*_*NDM-1*_ [85–87] and therefore, their detection in the KBTH in Ghana is of concern and calls for intensive surveillance and infections control measures to avert a possible outbreak. Also, the study report for the first time, the detection of ST164 OXA-58, NDM-1-producing *A. baumannii* strain in Ghana. ST164, NDM-1 positive *A. baumannii* strains are endemic in Thailand [83], nevertheless, this strain has also been reported in China, Egypt, Sudan and recently, in Denmark [54,87–89]. It is worth noting that the isolates described in these previous studies lacked the *bla*_*OXA-58*_ gene which was present in our isolate. ST164 *A. baumannii* is an emerging high-risk clone that needs to be controlled to avoid their spread. This study also identified ST2146 OXA-420 positive *A. baumannii* strain (Aba23). ST2146 is a novel sequence type, newly assigned to a CR-*A. baumannii* isolate carrying *bla*_*OXA-420*_ and *bla*_*NDM-1*_ in a previous study in Ghana. [12] and its detection in this present study indicates ST2146 may be an emerging clone steadily spreading in Ghana. Similarly, the detection of ST16 (isolate Aba08) and ST52 (Aba11) sequence types frequently associated with Asian countries such as Myanmar, Thailand and Vietnam [90,91] may suggest a possible emergence of these strains in Ghana.

Although only a limited number of genomes were studied due to financial constraints, the study nevertheless has revealed significant findings, that may shed light on the dynamics of CR and XDR in *A. baumannii* as well as added to the limited number of genomic characterisation studies of *A. baumannii* in Ghana and Africa.

## Conclusion

Our study highlights the occurrence of multiple carbapenemase-encoding genes, other AMR encoding genes such as aac(6’) aadA2, aph(3’)-19, 16SrRNAmethythransrase, ParC/gyrA, Sul1/Sul2, qacE delta1/qacG, mphE, and different efflux pumps as major antimicrobial mediating factors in XDR clinical *A. baumannii* isolates. The study also reveals the close association of carbapenemase-encoding genes with diverse transferable MGEs that could facilitate their spread among *A. baumannii* and other Gram-negative pathogens. We reported to the best of our knowledge, the first detection of high-risk clones ST2 OXA-23, NDM-1 producing *A. baumannii* and ST164 OXA-58, NDM-1-producing *A. baumannii* strains in Ghana. Our findings further emphasize the need for more genomic epidemiological studies and continuous surveillance to ensure the effective control of the spread of resistant organisms and high risk clones especially in hospitals.
